# Prevalence of Pretreatment HIV-1 Drug Resistance in Armenia in 2017–2018 and 2020–2021 following a WHO Survey

**DOI:** 10.3390/v14112320

**Published:** 2022-10-22

**Authors:** Alina Kirichenko, Dmitry Kireev, Ilya Lapovok, Anastasia Shlykova, Alexey Lopatukhin, Anastasia Pokrovskaya, Natalya Ladnaya, Trdat Grigoryan, Arshak Petrosyan, Tatevik Sarhatyan, Narina Sargsyants, Tamara Hovsepyan, Hovsep Ghazaryan, Hermine Hovakimyan, Siranush Martoyan, Vadim Pokrovsky

**Affiliations:** 1 Central Research Institute of Epidemiology, 111123 Moscow, Russia; 2 Department of Infectious Diseases with Courses of Epidemiology and Phthisiology, Medical Institute, Peoples’ Friendship University of Russia (RUDN University), 117198 Moscow, Russia; 3 National Center for Infectious Diseases, Yerevan 0025, Armenia

**Keywords:** HIV-1, PDR, drug resistance, EECA, Armenia

## Abstract

The increased antiretroviral therapy (ART) coverage of patients in the absence of routine genotyping tests and in the context of active labor migration highlight the importance of HIV-1 drug resistance (DR) surveillance in Armenia. We conducted a two-phase pretreatment DR (PDR) study in 2017–2018 (phase I; 120 patients) and 2020–2021 (phase II; 133 patients) according to the WHO-approved protocol. The analysis of HIV-1 genetic variants showed high degrees of viral diversity, with the predominance of A6. The prevalence of any PDR was 9.2% in phase I and 7.5% in phase II. PDR to protease inhibitors was found only in 0.8% in phase II. PDR to efavirenz and nevirapine was found among 5.0% and 6.7% of patients in phase I, and 6.0% and 6.8% of patients in phase II, respectively. The prevalence of PDR to nucleoside reverse-transcriptase inhibitors decreased from 5.0% in phase I to 0.8% in phase II. In addition, we identified risk factors associated with the emergence of DR—male, MSM, subtype B, and residence in or around the capital of Armenia—and showed the active spread of HIV-1 among MSM in transmission clusters, i.e., harboring DR, which requires the immediate attention of public health policymakers for the prevention of HIV-1 DR spread in the country.

## 1. Introduction

HIV infection is a major public health problem in Eastern Europe and Central Asia (EECA), where new infections continue to increase [1].

At the same time, one of the EECA countries, Armenia, is characterized by a relatively low HIV-1 infection prevalence rate among these regions, with 369 new HIV cases registered in 2020, and the total cumulative number of cases being 4154. [2]. The estimated proportion of people living with HIV in the national population is 0.12%; this percentage is doubled among ages 15–49 [1]. In 2017, Armenia implemented the “treat-all”, “treat-early”, and “treatment as the prevention” WHO policy, and there was significant progress in of care and treatment for people living with HIV (PLWH). The rate of people who knew their HIV-positive status notably increased from 48% in 2016 to 77% in 2020. The proportion of PLWH who received antiretroviral therapy (ART) from the total diagnosed PLWH expanded from 55% in 2016 to 81% in 2020. In addition, the percentage of PLWH receiving ART who achieved an undetectable HIV viral load also increased from 68% in 2016 to 86% in 2020 [3,4].

Current national HIV-infection treatment and prevention clinical guidelines in Armenia are in line with the updated WHO recommendations [5]. Since 2017, ART initiation has been offered to all people living with HIV following a confirmed HIV diagnosis, regardless of clinical stage and CD4 cell count [6].

However, a further increase in ART coverage without resistance testing is likely to be associated with an increase in HIV drug resistance (DR) and its spread, which can limit the success of ART [7,8,9].

Unfortunately, resistance testing prior to ART initiation or surveillance of pretreatment drug resistance (PDR) as recommended by the WHO [10] is not a common approach in Armenia, due to the unavailability of the procedure and its high costs [11]. By 2018, less than 100 nucleotide sequences of HIV from Armenian patients were available in public databases to assess HIV DR to major classes of antiretroviral (ARV) drugs. Over the past 10 years, a period in which the coverage of ART significantly increased, no studies assessing the prevalence of HIV DR in the country were conducted [12].

In Armenia, HIV is mainly transmitted through heterosexual contact (73% of the cumulative number of HIV cases), and 69% of all diagnosed HIV cases are men [2]. Notably, Armenia is characterized by numerous labor migrants, who are among the key populations most vulnerable to HIV in the country [13]. More than 50% of infections among Armenian citizens were shown to have occurred outside the country [14]. In addition, the high rate of migration to the Russian Federation, other parts of Eastern Europe, and Central or Western Asia for work could be a risk factor for further HIV transmission and DR.

In this regard, we conducted a two-phase PDR study according to the WHO guidelines. The results of the first phase, which was performed in six countries of the EECA region, including Armenia, were published [15].

Until 2019, the main first-line ART regimens were based on two nucleoside reverse-transcriptase inhibitors (NRTIs) (mainly, tenofovir disoproxil fumarate (TDF) (or zidovudine (AZT)) + lamivudine (3TC) (or emtricitabine (FTC)) and one non-nucleoside reverse-transcriptase inhibitor (NNRTI) (efavirenz (EFV) or nevirapine (NVP)) or dolutegravir (DTG) [16]. To date, Armenia has transitioned from NNRTI- to DTG-containing first-line ART regimens. Moreover, now, the preferred first-line ART regimens contain two NRTIs (mainly, TDF + 3TC (or FTC)) and DTG [17]. Notably, in 2016, Armenia put in place agreements to allow generic manufacturers to produce DTG. However, only in 2021, a significant proportion (more than 80%) of patients began to receive DTG; in 2020, the rate was about 30%, in 2019, just over 11%, and in 2018, less than 1% [6,18].

Thus, the continuation of our PDR study was performed to verify any changes in the features and prevalence of HIV DR among patients starting non-NNRTI ART regimens.

In this study, we aimed to assess PDR prevalence in 2020–2021, compare the new data with those obtained in the first phase of the study in 2017–2018, and clarify the factors associated with the emergence of drug resistance in Armenia.

## 2. Materials and Methods

### 2.1. Study Population

We conducted a two-phase, nationally representative, cross-sectional, retrospective surveillance study of PDR in 2017–2018 (phase I) and 2020–2021 (phase II) according to the WHO-approved protocol. The study population included adult patients (age 18 or over) eligible to initiate first-line ART, including ARV drug naïve people and people who had interrupted ART before restarting first-line ART without reported virological failure. Study enrollment took place at the National Center for Infectious Diseases (Yerevan, Armenia), which is the only ART delivery clinic in the country.

The number of patients starting first-line ART in the country was rather small, and all of them were enrolled in the study where informed written consent was obtained. In total, 143 patients and 164 patients initiating or reinitiating first-line ART in 2017–2018 (phase I) and 2020–2021 (phase II), respectively, were included in the study. Blood samples were collected for 6 months in each phase; between 1 October 2017 and 31 March 2018 in phase I, and between 1 October 2020 and 31 March 2021 in phase II. The collecting and processing of blood samples followed WHO laboratory guidance.

Demographic, clinical, and epidemiological data of the participants were obtained from the participants’ medical records.

### 2.2. RNA Extraction and HIV-1 Sequencing

An AmpliSens^®^ HIV-Resist-Seq kit (Central Research Institute of Epidemiology, Russia) was used for RNA extraction from blood plasma samples and for the amplification and sequencing of the HIV pol gene region encoding a protease and part of a reverse transcriptase (2253–3369 bp according to the HXB-2 strain; GenBank accession number: K03455).

Quality assurance of HIV-1 sequences was carried out using the WHO HIV DR quality control tool (http://pssm.cfenet.ubc.ca/who_qc/, accessed on 6 July 2022) before data analysis.

The HIV-1 sequences generated from this study were submitted to the NCBI database.

### 2.3. HIV-1 Subtyping

HIV-1 subtypes were determined using Stanford HIV Resistance Database (https://hivdb.stanford.edu/, accessed on 6 July 2022) and the HIV BLAST tool (https://www.hiv.lanl.gov/content/sequence/BASIC_BLAST/basic_blast.html, accessed on 6 July 2022) and were subsequently clarified via phylogenetic analyses.

Phylogenetic analyses were performed with MEGA 6.0 software using the maximum likelihood (ML) method with bootstrap (500 replications) and the generalized time reversible (GTR + G + I) model of nucleotide substitution with HIV-1 subtype references from Los Alamos National Laboratory (https://www.hiv.lanl.gov/content/index, accessed on 21 September 2022).

### 2.4. HIV-1 Drug-Resistance Analysis

The Stanford HIV Drug Resistance Database (HIVdb Program v 9.1) was used to describe and interpret the HIV DR level and drug-resistant mutations (DRMs), including the WHO’s surveillance DRMs (SDRMs) [19].

The DR level was classified according to the Stanford Penalty Score as high (60), intermediate (30–59), or low (15–29).

Sequences with PDR were defined as sequences with a Stanford Penalty Score of 15 or higher with respect to the following antiretroviral drugs according to the PDR criterion suggested by the WHO [20]:

- Non-nucleoside reverse-transcriptase inhibitors (NNRTIs): efavirenz (EFV) and nevirapine (NVP);
- Any nucleoside reverse-transcriptase inhibitors (NRTIs): abacavir (ABC), zidovudine (AZT), stavudine (d4T), didanosine (ddI), emtricitabine (FTC), lamivudine (3TC), and tenofovir disoproxil fumarate (TDF);
- Protease inhibitors (PIs): atazanavir/ritonavir (ATV/r), darunavir/ritonavir (DRV/r), and lopinavir/ritonavir (LPV/r).

### 2.5. Cluster Analysis and Tree Vizualization

Transmission clusters were determined by Cluster Picker 1.2.3 [21], with a genetic distance threshold of 0.045 and bootstrap support of more than 0.9. Tree visualization and annotation were performed using iTOL (https://itol.embl.de/, accessed on 22 September 2022).

### 2.6. Statistical Analysis

Estimates of the prevalence of PDR were calculated with 95% confidence intervals (CIs). Comparisons among groups were performed using Fisher’s exact tests. To determine the significance of the difference between the means of two unrelated groups, independent *t*-tests were used. Statistical significance was defined as *p*-values < 0.05. All analyses were performed using STATA (v15).

### 2.7. Ethics

This study was approved by the local ethics committee of the Central Research Institute of Epidemiology (Moscow, Russian Federation) and the ethics review committee of the National Center for Infectious Diseases (Yerevan, Armenia) according to national legislation. The informed written consent of each patient who participated in the study was obtained prior to the sampling and collection of clinical, demographic, and epidemiological data. All the data were anonymized and coded at a national level.

## 3. Results

### 3.1. Characteristics of the Study Cohorts

The total specimens eligible for genotyping were 143 and 164 for phases I and II, respectively. The genotyping results were successfully obtained for 132 and 148 of these. The total sequences subjected to quality control using the WHO HIV DR quality-control tool were 120 and 133. Thus, the overall efficiencies were 83.9% and 81.1% for phases I and II, respectively.

The epidemiological and clinical data of participants from both sampling phases in 2017–2018 (I) and 2020–2021 (II) are summarized and compared in Table 1.

The median ages of the patients were similar in both phases of the study, ranging from 37 to 41.

Over half of the patients were male (65.8% and 69.2%) and reported heterosexual contact (85.0% and 75.9%) as the main risk factor in phases I and II, respectively. The prevalence of the MSM risk factor among patients in phase II was significantly higher than in phase I (5.0% vs. 13.5%; *p* = 0.03).

The median CD4+ T-cell count decreased from 325 cells/mm^3^ to 216 cells/mm^3^ in the first and second phases, respectively, but this difference was not statistically significant (*p* = 0.53).

The majority of patients in both phases had no antiretroviral (ARV) exposure and had their first positive immune blot in 2017 in phase I (71.7%) and between 2020 (33.8%) and 2021 (59.4%) in phase II. Thus, most patients were newly diagnosed.

### 3.2. Prevalence of HIV-1 Genetic Variants

**Figure 1:**
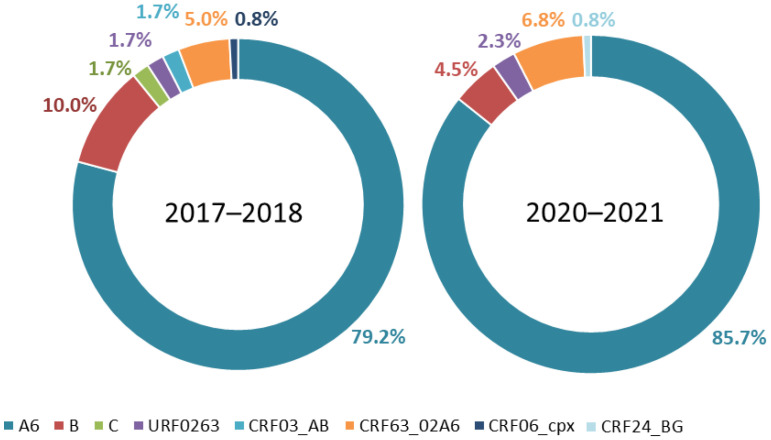
Prevalence of HIV-1 genetic variants in Armenia in the 2017–2018 and 2020–2021 sampling years.

A high degree of viral diversity was observed (Figure 1). The most prevalent HIV-1 genetic variant in both phases was sub-subtype A6, with 79.2% (95% CI, 63.2–95.1%) and 85.7% (95% CI, 70.0–101.4%) in 2017–2018 and 2020–2021 (*p* = 0.1867), respectively. The temporal trends of sub-subtype A6 increased, whereas subtype B had a decreasing trend. HIV infections in Armenia were caused by subtype B in 10.0% (95% CI, 5.2–17.5%) and 4.5% (95% CI, 1.7–9.8%) of cases in phases I and II (*p* = 0.14), respectively. HIV-1 recombinant forms were also identified. CRF63_02A6 was found in 5.0% and 6.8% of cases in phases I and II, respectively. In five other samples (1.7% in phase I and 2.3% in phase II) a unique recombinant form (URF) was identified, genetically close to the AG recombinant forms isolated in Uzbekistan (in particular, sample AY829204), which is currently regarded as a recombinant formed by CRF63_02A6 and CRF02_AG (URF0263). In addition, we identified two samples of CRF03_AB (phase I), one sample of CRF24_BG (phase II), and one of CRF06_cpx (phase I), which is rare in the EECA region.

### 3.3. Prevalence of DRMs

The most prevalent PI DRMs were M46I (2.5%) and L10F (1.7%) in phase I, and these were found in one person (0.8%) in phase II.

The most frequent NRTI DRM was A62V, with prevalence rates of 28.3% and 24.1% in phases I and II, respectively. Of note, this is a polymorphic mutation in sub-subtype A6 and, alone, probably confers little to no NRTI resistance. T69D and L210W were also found in 2.5% of patients in phase I.

Of the NNRTI DRMs, E138A (4.2% and 8.3%) and V106I (5.8% and 3.8%) were the most frequently observed in phases I and II, respectively. Other substitutions were found in codon 138 (E138G/K), with a prevalence of 3.8% among patients in phase II. Mutations in codon 138 were not considered for PDR estimation, because they are associated with reduce susceptibility to rilpivirine. In addition, E138A is a polymorphic mutation in sub-subtype A6.

Notably, the prevalence rates of K103N, which is important for both the clinical impact and surveillance of DR transmission because of its long persistence [22], were 1.7% and 3.0% in phases I and II, respectively.

The frequencies of all DRMs are shown in Table 2.

### 3.4. Prevalence of PDR

The prevalence of any PDR slightly decreased from 9.2% (95% CI, 4.6–16.4%) in 2017–2018 to 7.5% (95% CI, 3.6–13.8%) in 2020–2021 (*p* = 0.66).

**Figure 2:**
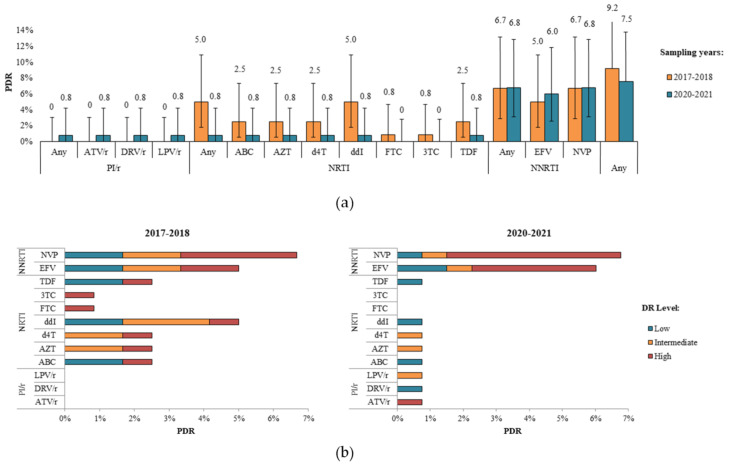
PDR prevalence in Armenia in the 2017–2018 and 2020–2021 sampling years for (**a**) antiretroviral drugs and (**b**) antiretroviral drugs with DR levels. Error bars represent 95% CIs. Abbreviations: ATV/r, atazanavir/ritonavir; DRV/r, darunavir/ritonavir; LPV/r, lopinavir/ritonavir; ABC, abacavir; AZT, zidovudine; d4T, stavudine; ddI, didanosine; FTC, emtricitabine; 3TC, lamivudine; TDF, tenofovir disoproxil fumarate; EFV, efavirenz; NVP, nevirapine.

PDR to PIs was found only in one patient (0.8% (95% CI, 0.0–4.2%)) in phase II of the study (Figure 2a).

PDR to NRTIs decreased from 5.0% (95% CI, 1.8–10.9%) in phase I to 0.8% (95% CI, 0.0–4.2%) in phase II (*p* = 0.06), mainly due to ddI. The PDR prevalence rates of other NRTI ARV drugs were 2.5% (95% CI, 0.5–7.3%) and 0.8% (95% CI, 0.0–4.2%) for ABC, AZT, d4T, and TDF, and 0.8% (95% CI, 0.0–4.6%) and 0.0% (95% CI, 0.0–2.8%) for FTC and 3TC in phases I and II, respectively.

PDR to NNRTIs slightly increased from 5.0% (95% CI, 1.8–10.9%) in phase I to 6.0% (95% CI, 2.6–11.8%) in phase II for EFV (*p* = 0.79) and remained almost the same in phases I and II (6.7% (95% CI, 2.9–13.1%) and 6.8% (95% CI, 3.1–12.8%)) for NVP. However, PDR in patients in phase II of the study was predominantly found to be at high levels (Score ≥ 60) (Figure 2b).

### 3.5. Factors Associated with PDR

We explored PDR prevalence and its associated risk factors—sex, transmission risk group, HIV-1 genetic variant, prior ARV exposure, and region of origin.

Because of the small sample size, most of the identified associations were not statistically significant; therefore, we combined the data obtained in the two phases for this analysis. The associations in each phase are separately and collectively presented in Table 3.

There was a significant correlation between sex and DR. Men were more likely to have HIV drug-resistant variants (11.7%) than women (2.4%) (*p* = 0.0156). Among the risk groups, the MSM group had the highest prevalence of DR (4/24; 16.7%), which was not significant due to the small sample size (*p* = 0.1233).

There was a significant difference in the prevalence of PDR among HIV-1 genetic variants. In total, 10 cases of sub-subtype A6 out of 209 (4.8%) and 6 cases of subtype B out of 18 (33.3%) showed PDR (*p* = 0.0005).

We also analyzed the association between PDR and patients’ regions of origin. As a result, PDR was found in residents of the capital, Yerevan (12.5%; *p* = 0.0143), and within 50 km of it (14.1%; *p* = 0.0026) more often than in residents of regions more than 50 km away (1.9%).

There were no significant associations between the emergence of PDR in patients and their experience with ART.

### 3.6. Cluster Analysis

**Figure 3:**
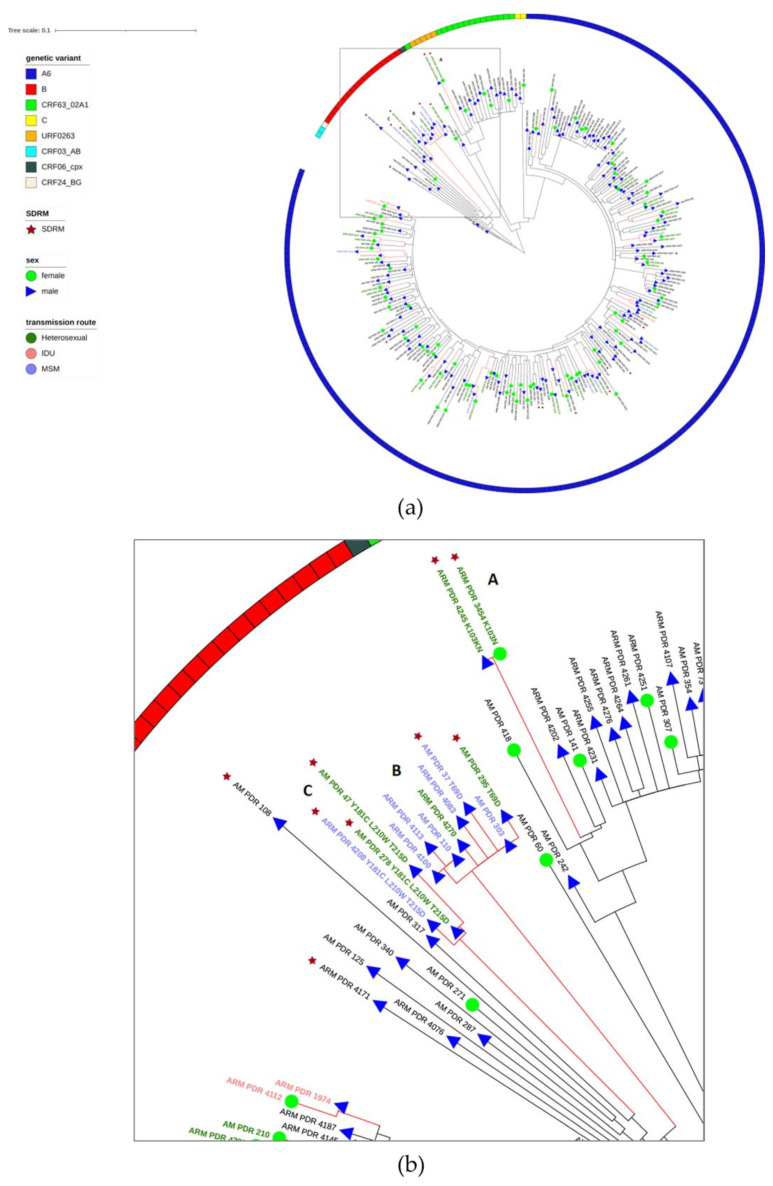
The maximum likelihood (ML) tree of the pol region sequences from both phases of study (**a**), with three clusters (A, B, and C) harboring shared surveillance drug resistant mutations (SDRMs) (**b**). Genetic variants are displayed on the outside of the tree (colored ring). Red branches indicate transmission clusters. Green circles indicate women, and blue triangles are men. Red stars indicate sequences harboring SDRMs. Label branches of clustering sequences harboring SDRMs are contained in corresponding SDRMs. Labels are colored according to the transmission route: green, pink, and blue for heterosexual, IDU, and MSM, respectively. Abbreviations: MSM, men having sex with men; IDU, intravenous drug users.

To further understand the transmission of DR, we analyzed transmission clusters based on HIV-1 sequences from both phases of the study and showed these on the phylogenetic tree (Figure 3a). A total of 66/253 (26.1%) sequences were linked, forming 26 transmission clusters ranging in size from two to eight individuals.

We determined the characteristics of the clusters, including phase of study, sex, transmission route, region of origin, genetic variant, and SDRMs (Table 4).

The proportion of patients from phase II entering the transmission clusters was higher (44/133, 33.1%) than patients from phase I (22/120, 18.3%) (*p* = 0.0097). Notably, there were six clusters that included patients from both phases of study, five clusters that contained patients only from first phase, and 15 clusters from second only.

We found that MSM patients were significantly more often involved in transmission clusters (14/24, 58.3%; *p* = 0.0001), whereas heterosexual patients were less often involved (47/203, 23.2%; *p* = 0.0468).

Interestingly, we found that among clustering male individuals, MSM and heterosexual contact were cross-linked. Five clusters involving only 19 male patients included at least one MSM in each of them.

Individuals infected with the B subtype were part of transmission clusters more often (11/18, 61.1%) than those infected with other genetic variants (55/235, 23.4%) (*p* = 0.0012).

The frequency of SDRMs did not significant differ (*p* = 0.2822) between clustering (7/66, 10.6%) and non-clustering individuals (12/187, 6.4%). We also observed in the network three clusters with shared SDRMs (Figure 3b). Cluster A contained a man and woman from phase II of the study infected with URF0263 with the K103N (NNRTI mutation). Cluster B included four male patients (3 MSM + 1 heterosexual) from phase I and four male patients (3 MSM + 1 heterosexual) from phase II infected with subtype B, two of them harboring T69D (NRTI mutation). Cluster C contained two heterosexual male individuals from phase I and one MSM male individual from phase II infected with subtype B with L210W, T215D (NRTI mutations), and Y181C (NNRTI mutation).

Among the analyzed factors, no significant differences were found between clustering and non-clustering individuals in region of origin and sex. However, in clusters containing more than two individuals (five clusters), the difference between involved men (21/171, 12.3%) and women (3/82, 3.7%) was significant (*p* = 0.0371).

## 4. Discussion

Every year, Armenia nears the targets to end the public health threat of the AIDS epidemic. Based on data from national studies [3,4], there are gaps in the continuum of care and treatment for people living with HIV in terms of approaching the first (77%) and second (81%) “95” targets. The third “95” (86%) is the closest to being achieved.

It is interesting to note that although the inclusion criteria were the same in both phases of the study, in the second phase, there was a three-fold increase in MSM (*p* = 0.03). An increase in HIV prevalence among MSM in Armenia from 0.8% to 5.0% in 2016–2021 was also shown in an integrated biological and behavioral assessment report [23]. In addition, in the second phase, a 1.5-fold decrease in CD4+ T-cell count (*p* = 0.53) can be seen, which likely indicates a higher number of late-diagnosed patients. In our study, it is difficult to understand the reasons for this; however, it may be related with delaying and/or avoiding seeking medical care due to the COVID-19 pandemic.

In 2017, Armenia adopted the “treat-all”, “treat-early”, and “treatment as the prevention” WHO strategy; as a result, the coverage of patients who received ART significantly increased, which led to an increase in the prevalence of HIV-1 DR.

This study demonstrated a moderate prevalence of DR among newly diagnosed PLWH before initiation or re-initiation of first-line ART in Armenia. It was higher than the prevalence (1.5%) determined in the country in a previous study [24] of 67 treatment-naïve patients whose blood samples were collected in 2009–2010.

In the present study, the prevalence of any PDR ranged from 9.2% in 2017–2018 (phase I) to 7.5% in 2020–2021 (phase II). Notably, PDR to NNRTIs EFV and NVP was found among 5.0% and 6.7% of patients in phase I, and 6.0% and 6.8% of patients in phase II, respectively. Thus, the WHO-recommended 10% threshold at which it is necessary to change the ART regimen [10,20] was not reached. It should be noted that there was no increase in the prevalence of DR over time. Moreover, because of the current preferred non-NNRTI first-line ART regimen, it can be expected that the prevalence of resistance among patients initiating ART will not increase.

The current study was limited by the lack of the analysis of DR to integrase inhibitors, although the first-line ART regimen at that time already included dolutegravir. From our study, only the predicted efficacy of the NRTI components of the recommended ART regimen could be concluded. DR to tenofovir (0.8–2.5%), lamivudine (0–0.8%), and emtricitabine (0–0.8%) was found in isolated patients.

Because of the high percentage of PLWH with successful ART and low prevalence of DR to first-line ART drugs, we suggest that the genotyping test is not required as routine care prior to ART in Armenia. However, it is periodically necessary to conduct surveillance studies in this country, which should include the analysis of DR to integrase inhibitors.

This is especially important due to the high level of population migration, the main risk factor for HIV infection in the country [13]. The high level of population migration is reflected in the high diversity of HIV genetic variants circulating in the country. Armenia has the largest number of HIV subtypes and CRFs among the EECA countries according to the results of our first study [15].

The predominance of HIV-1 sub-subtype A6 in Armenia is consistent with previously published data on the circulation of HIV-1 genetic variants in the former Soviet Union [15,25,26] and suggests a close relationship between the HIV epidemics in these countries. Since the Russian Federation is historically the main state for Armenian labor immigrants to work in [13,24], it is quite natural that A6 dominates in both countries. The closeness of the epidemiological process in Armenia and the Russian Federation is also confirmed by the similar prevalence of CRF63_02A6, which accounts for 7.0% in Russia [27] and ranges from 5.0% to 6.8% in Armenia according to our results. At the same time, in the countries of Central Asia, the prevalence of CRF63_02A6 is much higher, and in Tajikistan, it is the dominant genetic variant [15]. Five samples, identified as URF0263, indicated a connection between the HIV-1 epidemics in Armenia and Central Asian countries, where various AG recombinants are most often detected [28]. However, all changes in the distribution of genetic viral variants were not significant, perhaps due to the small size of the studied groups. For more robust molecular epidemiology studies, sampling density should be increased.

Our results cast a light on the factors associated with PDR in Armenian PLWH. The rate of PDR was the highest among men (11.7%), subtype B (33.3%), and residents of the capital, Yerevan (12.5%), and living less than 50 km from it (14.1%). In addition, the MSM transmission risk group had the highest prevalence of DR (16.7%), which was not significant due to the small sample size. These findings should be validated using a larger sample size.

Cluster analysis allowed us to better understand the features of HIV distribution and DR transmission. The larger clusters, which consisted of more than two sequences, included mostly men (*p* = 0.029). In addition, large growing clusters, which consisted of sequences from both phases, always included samples from MSM. The cross-linked sequences from MSM and heterosexual men in the absence of women sequences in clusters were common. We can conclude that homosexual transmission cases of infection are underreported. Additionally, MSM is a risk group in which the fastest spread of infection with drug-resistant viral variants is taking place.

The results of our analyses suggest the necessity of implementing effective interventions targeting MSM of PLWH in Armenia to prevent the spread of HIV-1 DR.

It is important to note that the sample mainly consisted of newly diagnosed patients. Thus, the described prevalence of DR and genetic variants reflected the current situation in Armenia.

The analysis of DR was performed at a significant time point, i.e., after the large-scale introduction of ART at the time of the transition to new first-line regimens.

In conclusion, our work expanded the available molecular and epidemiological data from Armenian HIV-infected patients by three-fold. We believe that this is important for a public-health response to the HIV epidemic, including the development of antiviral drugs and vaccines, which are especially essential in a country without routine HIV DR testing.

## Figures and Tables

**Table 1:** Epidemiological and clinical characteristics of the study population.

Characteristics	Sampling Years		*p*-Value
	2017–2018	2020–2021	
**Number of patients**	120	133	
**Median age, years (IQR)**	37 (29–45)	41 (30–47)	0.56
**Sex, n (%)**			
Male	79 (65.8)	92 (69.2)	0.59
Female	41 (34.2)	41 (30.8)	0.59
**Transmission risk group, n (%)**			
Heterosexual	102 (85.0)	101 (75.9)	0.08
MSM	6 (5.0)	18 (13.5)	**0.03**
IDU	11 (9.2)	14 (10.5)	0.83
Unknown	1 (0.8)	0	-
**Viral load (log10 copies/mL), median (IQR)**	5.2 (4.3–5.9)	5.0 (4.5–5.6)	0.48
**CD4+ T-cell count (cells/mm^3^), median (IQR)**	325 (78–504)	216 (62–409)	0.53
**Prior ARV drug exposure, n (%)**			
Yes	7 (5.8)	6 (4.5)	0.78
No	113 (94.2)	127 (95.5)	0.78
**Date of first positive immune blot, n (%)**			
2007	1 (0.8)	0	-
2009	1 (0.8)	0	-
2010	1 (0.8)	0	-
2011	3 (2.5)	0	0.11
2012	3 (2.5)	2 (1.5)	0.67
2013	1 (0.8)	0	-
2014	5 (4.2)	1 (0.8)	0.10
2015	3 (2.5)	1 (0.8)	0.35
2016	4 (3.3)	1 (0.8)	0.19
2017	86 (71.7)	0	**0.0001**
2018	7 (5.8)	0	**0.0001**
2019	NA	4 (3.0)	-
2020	NA	45 (33.8)	-
2021	NA	79 (59.4)	-
Unknown	5 (4.2)	0	**0.02**

Statistically significant values (*p* < 0.05) are indicated in bold. Abbreviations: IQR, interquartile range; MSM, men having sex with men; IDU, intravenous drug users; ARV, antiretroviral; NA, not applicable.

**Table 2:** Prevalence of DRMs in Armenia in 2017–2018 and 2020–2021.

Sampling Data	2017–2018 (n = 120), %	2020–2021 (n = 133), %	*p*-Value
PI DRMs			
L10F	1.7	0.8	0.61
L24F	0	0.8	-
K43T	0	0.8	-
**M46I**	2.5	0.8	0.35
M46V	0	0.8	-
**G73S**	0	0.8	-
T74P	0.8	0.8	-
T74S	0.8	0	-
**I84V**	0	0.8	-
NRTI DRMs			
E40F	0.8	0	-
**M41L**	0.8	0	-
E44D	0	1.5	0.50
A62V	28.3	24.1	0.48
**D67N**	0.8	0	-
**T69D**	2.5	0	0.11
T69N	0.8	0	-
**K70R**	0.8	0	-
**V75A**	0.8	0	-
**M184V**	0.8	0	-
**L210W**	2.5	0.8	0.35
**T215D**	1.7	0.8	0.61
**T215Y**	0.8	0	-
**K219E**	0.8	0	-
NNRTI DRMs			
A98G	1.7	0	0.22
**K101E**	0	1.5	0.50
**K103N**	1.7	3.0	0.69
V106I	5.8	3.8	0.56
V108I	2.5	0.8	0.35
E138A	4.2	8.3	0.21
E138G	0	3.0	0.12
E138K	0	0.8	-
V179D	0.8	0	-
**Y181C**	1.7	0.8	0.61
Y181S	0	0.8	-
**G190A**	0.8	0.8	-
K238T	0	0.8	-

Surveillance drug resistant mutations are in bold. Abbreviations: DRMs, drug resistant mutations; PI, protease inhibitor; NRTI, nucleoside reverse-transcriptase inhibitor; NNRTI, non-nucleoside reverse-transcriptase inhibitor.

**Table 3:** Prevalence of PDR in the study population stratified by different groups.

Phases	I		II		I + II	
Characteristics	Total, n	PDR, n (%)	Total, n	PDR, n (%)	Total, n	PDR, n (%)
Sex						
Male	79	10 (12.7)	92	10 (10.8)	171	20 (11.7)
Female	41	1 (2.4)	41	1 (2.4)	82	2 (2.4)
Transmission risk group						
Heterosexual	102	9 (8.8)	101	6 (5.9)	203	15 (7.4)
MSM	6	1 (16.7)	18	3 (16.7)	24	4 (16.7)
IDU	11	1 (9.1)	14	1 (7.1)	25	2 (8)
Unknown	1	0	0	0	1	0
HIV-1 genetic variant						
A6	95	4 (4.2)	114	6 (5.3)	209	10 (4.8)
B	12	5 (41.7)	6	1 (16.7)	18	6 (33.3)
Non-A (Including B)	25	7 (28.0)	19	4 (21.1)	44	11 (25.0)
Prior ARV exposure						
Yes	7	0	6	2 (33.3)	13	2 (15.4)
No	113	11 (9.7)	127	8 (6.2)	240	19 (7.9)
Region of origin						
Yerevan	30	6 (20.0)	34	2 (5.9)	64	8 (12.5)
<50 km from Yerevan	34	5 (14.7)	44	6 (13.6)	78	11 (14.1)
>50 km from Yerevan	46	0	55	2 (3.6)	101	2 (2.0)

Abbreviations: MSM, men having sex with men; IDU, intravenous drug users; ARV, antiretroviral.

**Table 4:** Factors associated with transmission within clusters.

Characteristics	Total, n	Clustering, n (%)
Phase		
I	120	22 (18.3)
II	133	44 (33.1)
Sex		
Male	171	44 (25.7)
Female	82	22 (26.8)
Transmission risk group		
Heterosexual	203	47 (23.2)
MSM	24	14 (58.3)
IDU	25	5 (20.0)
Unknown	1	0
HIV-1 genetic variant		
A6	209	53 (25.4)
B	18	11 (61.1)
C	2	0
URF0263	5	2 (40.0)
CRF03_AB	2	0
CRF06_cpx	1	0
CRF24_BG	1	0
CRF63_02A1	15	0
Region of origin		
Yerevan	64	19 (29.7)
<50 km from Yerevan	78	15 (19.2)
>50 km from Yerevan	101	32 (31.7)

Abbreviations: MSM, men having sex with men; IDU, intravenous drug users.

## Data Availability

The HIV-1 sequences generated from this study are available in the NCBI database, with GenBank accession numbers MW484200–MW484319 (phase I) and OP009620–OP009752 (phase II).

## References

[1] Joint United Nations Programme on HIV/AIDS 2021. https://www.unaids.org/sites/default/files/media_asset/JC3032_AIDS_Data_book_2021_En.pdf.

[2] European Centre for Disease Prevention and Control/WHO Regional Office for Europe (2021). HIV/AIDS surveillance in Europe 2021–2020 data. Stockholm: ECDC. https://www.ecdc.europa.eu/en/publications-data/hiv-aids-surveillance-europe-2021-2020-data.

[3] European Centre for Disease Prevention and Control (2018). Continuum of HIV care. Monitoring Implementation of the Dublin Declaration on Partnership to Fight HIV/AIDS in Europe and Central Asia: 2018 Progress Report. Stockholm: ECDC. https://www.ecdc.europa.eu/en/publications-data/continuum-hiv-care-monitoring-implementation-dublin-declaration-2018-progress.

[4] European Centre for Disease Prevention and Control (2021). Continuum of HIV Care. Monitoring Implementation of the Dublin Declaration on Partnership to Fight HIV/AIDS in Europe and Central Asia: 2020 Progress Report. Stockholm: ECDC. https://www.ecdc.europa.eu/en/publications-data/hiv-continuum-care-monitoring-implementation-dublin-declaration.

[5] World Health Organization (2021). Consolidated Guidelines on HIV Prevention, Testing, Treatment, Service Delivery and Monitoring: Recommendations for a Public Health Approach. https://www.who.int/publications/i/item/9789240031593.

[6] International Treatment Preparedness Coalition Monitoring the Procurement of Drugs for the Treatment of HIV Infection and HCV. Development of Solutions to Optimize the Situation in Order to Promote Uninterrupted Access to Drugs in the Republic of Armenia, 2018-2019. https://itpc-eeca.org/wp-content/uploads/2019/11/Monitoring-zakupok-preparatov-Armeniya_2018-2019.pdf.

[7] Wittkop L., Günthard H.F., de Wolf F., Dunn D., Cozzi-Lepri A., de Luca A., Kücherer C., Obel N., von Wyl V., Masquelier B. (2011). Effect of transmitted drug resistance on virological and immunological response to initial combination antiretroviral therapy for HIV (EuroCoord-CHAIN joint project): A European multicohort study. Lancet Infect. Dis..

[8] Macdonald V., Mbuagbaw L., Jordan M.R., Mathers B., Jay S., Baggaley R., Verster A., Bertagnolio S. (2020). Prevalence of pretreatment HIV drug resistance in key populations: A systematic review and meta-analysis. J. Int. AIDS Soc..

[9] Hamers R.L., Schuurman R., Sigaloff K.C., Wallis C.L., Kityo C., Siwale M., Mandaliya K., Ive P., Botes M.E., Wellington M. (2012). Effect of pretreatment HIV-1 drug resistance on immunological, virological, and drug-resistance outcomes of first-line antiretroviral treatment in sub-Saharan Africa: A multicentre cohort study. Lancet Infect. Dis..

[10] World Health Organization (2017). Guidelines on the Public Health Response to Pretreatment HIV Drug Resistance. https://apps.who.int/iris/bitstream/handle/10665/255880/9789241550055-eng.pdf?sequence=1&isAllowed=y.

[11] Gokengin D., Oprea C., Begovac J., Horban A., Zeka A.N., Sedlacek D., Allabergan B., Almamedova E.A., Balayan T., Banhegyi D. (2018). HIV care in Central and Eastern Europe: How close are we to the target?. Int. J. Infect. Dis..

[12] Amangaldiyeva A., Davlidova S., Baiserkin B., Dzissyuk N., DeHovitz J., Ali S. (2019). Implementation of antiretroviral therapy (ART) in former Soviet Union countries. AIDS Res. Ther..

[13] Zhao F., Benedikt C., Wilson D. (2020). Tackling the World’s Fastest-Growing HIV Epidemic: More Efficient HIV Responses in Eastern Europe and Central Asia. Human Development Perspectives.

[14] Pokrovskaya A.V., Yumaguzin V.V., Kireev D.E., Vinnik M.V., Pokrovsky V.V. (2019). The Impact of Migration on HIV Infection Situation (Analytical Review). Vestn. Ross. Akad. Med. Nauk..

[15] Kirichenko A., Kireev D., Lopatukhin A., Murzakova A., Lapovok I., Saleeva D., Ladnaya N., Gadirova A., Ibrahimova S., Safarova A. (2022). Prevalence of HIV-1 drug resistance in Eastern European and Central Asian countries. PLoS ONE.

[16] Clinical Guidelines for the Treatment and Prevention of HIV Infection Using Antiretroviral Drugs. Yerevan, Republican Center for AIDS Prevention. 2017. p. 364. https://www.moh.am/uploads/Draft_ART%20GUIDE_24%2007%2017%20(2).pdf.

[17] Ministry of Health of the Republic of Armenia Order No. 3904-A dated 25 December 2019. Amendments to Orders Dated 7 August 2017 No. 2429-A and 8 November 2017 No. 3225-A. https://www.moh.am/uploads/3904.pdf.

[18] International Treatment Preparedness Coalition (2020). Monitoring the Procurement of Drugs for the Treatment of HIV Infection and HCV. https://docs.google.com/viewerng/viewer?url=https://itpc-eeca.org/wp-content/uploads/2021/12/2021_armenia_arv-procurement-monitoring_final-1.pdf&hl=ru.

[19] Bennett D.E., Camacho R.J., Otelea D., Kuritzkes D.R., Fleury H., Kiuchi M., Heneine W., Kantor R., Jordan M.R., Schapiro J.M. (2009). Drug resistance mutations for surveillance of transmitted HIV-1 drug-resistance: 2009 update. PLoS ONE.

[20] World Health Organization (2014). Surveillance of HIV Drug Resistance in Adults Initiating Antiretroviral Therapy (Pre-Treatment HIV Drug Resistance). https://www.who.int/publications/i/item/9789241507196.

[21] Ragonnet-Cronin M., Hodcroft E., Hue S., Fearnhill E., Delpech V., Brown A.J., Lycett S., UK HIV Drug Resistance Database (2013). Automated analysis of phylogenetic clusters. BMC Bioinform..

[22] Little S.J., Frost S.D., Wong J.K., Smith D.M., Pond S.L., Ignacio C.C., Parkin N.T., Petropoulos C.J., Richman D.D. (2008). Persistence of transmitted drug resistance among subjects with primary human immunodeficiency virus infection. J. Virol..

[23] National center for infectious diseases (2021). Integrated Bio-Behavioral Surveillance Surveys and Key Population Size Estimations among People Who Inject Drugs, Female Sex Workers, Men Who Have Sex With Men, and Transgender Persons.

[24] Laga V., Vasilyev A., Lapovok I., Grigoryan S., Papoyan A., Glushchenko N., Kazennova E., Bobkova M. (2015). HIV Type 1 Subtype A1 Dominates in Armenia. Cur. HIV Res..

[25] Bobkova M. (2013). Current status of HIV-1 diversity and drug resistance monitoring in the former USSR. AIDS Rev..

[26] Foley B.T., Leitner T., Paraskevis D., Peeters M. (2016). Primate immunodeficiency virus classification and nomenclature: Review. Infect. Genet. Evol..

[27] Lapovok I.A., Lopatukhin A.E., Kireev D.E., Kazennova E.V., Lebedev A.V., Bobkova M.R., Kolomeets A.N., Turbina G.I., Shipulin G.A., Ladnaya N.N. (2017). Molecular epidemiological analysis of HIV-1 variants circulating in Russia in 1987–2015. Ter. Arkh..

[28] Kostaki E.G., Karamitros T., Bobkova M., Oikonomopoulou M., Magiorkinis G., Garcia F., Hatzakis A., Paraskevis D. (2018). Spatiotemporal Characteristics of the HIV-1 CRF02_AG/CRF63_02A1 Epidemic in Russia and Central Asia. AIDS Res. Hum. Retrovir..

